# How Mycorrhizal Associations Influence Orchid Distribution and Population Dynamics

**DOI:** 10.3389/fpls.2021.647114

**Published:** 2021-05-07

**Authors:** Taiqiang Li, Shimao Wu, Wenke Yang, Marc-André Selosse, Jiangyun Gao

**Affiliations:** ^1^Yunnan Key Laboratory of Plant Reproductive Adaptation and Evolutionary Ecology, Yunnan University, Kunming, China; ^2^Laboratory of Ecology and Evolutionary Biology, Yunnan University, Kunming, China; ^3^Institut de Systématique, Évolution, Biodiversité, UMR 7205, CNRS, MNHN, UPMC, EPHE, Muséum National d’Histoire Naturelle, Sorbonne Universités, Paris, France; ^4^Department of Plant Taxonomy and Nature Conservation, Faculty of Biology, University of Gdańsk, Gdańsk, Poland

**Keywords:** orchid mycorrhizal fungi, orchid performance, complementary selection, environmental variables, evolutionary constraints, mycorrhizal networks, keystone taxa

## Abstract

Orchid distribution and population dynamics are influenced by a variety of ecological factors and the formation of holobionts, which play key roles in colonization and ecological community construction. Seed germination, seedling establishment, reproduction, and survival of orchid species are strongly dependent on orchid mycorrhizal fungi (OMF), with mycorrhizal cheating increasingly observed in photosynthetic orchids. Therefore, changes in the composition and abundance of OMF can have profound effects on orchid distribution and fitness. Network analysis is an important tool for the study of interactions between plants, microbes, and the environment, because of the insights that it can provide into the interactions and coexistence patterns among species. Here, we provide a comprehensive overview, systematically describing the current research status of the effects of OMF on orchid distribution and dynamics, phylogenetic signals in orchid–OMF interactions, and OMF networks. We argue that orchid–OMF associations exhibit complementary and specific effects that are highly adapted to their environment. Such specificity of associations may affect the niche breadth of orchid species and act as a stabilizing force in plant–microbe coevolution. We postulate that network analysis is required to elucidate the functions of fungal partners beyond their effects on germination and growth. Such studies may lend insight into the microbial ecology of orchids and provide a scientific basis for the protection of orchids under natural conditions in an efficient and cost-effective manner.

## Introduction

Mycorrhizal associations play a key role in generating and maintaining plant diversity. Such associations not only enhance the acquisition, transmission, and cycling of nutrients in plants, but also mediate interactions among different plants and between plants and non-mycorrhizal fungi (Tedersoo et al., 2020). A growing body of research suggests that mycorrhizal symbionts are important drivers of biogeographic patterns, distributions, community dynamics, and the health of plants, mediated by their effects on dispersal and coexistence of species (Delavaux et al., 2019; Trivedi et al., 2020). For example, ectomycorrhizae (EcM) interact with pathogenic fungi to maintain community diversity (Chen et al., 2019a). The family Orchidaceae is extremely diverse (with 28,000+ species), widely distributed across highly heterogeneous microenvironments, and exhibits large spatiotemporal variation in population size (Givnish et al., 2016; Fay, 2018; Djordjević and Tsiftsis, 2020). A common feature of all orchids is their obligatory dependence on orchid mycorrhizal fungi (OMF), which makes the presence of suitable OMF or co-dispersal with partners a prerequisite for the establishment and maintenance of orchid populations (Dearnaley et al., 2012; McCormick and Jacquemyn, 2014). Mycorrhizal symbiosis is especially important for plants associated with OMF and EcM, as they often have high mycorrhizal specificity (Põlme et al., 2018). Most species of EcM fungi exhibit short-distance dispersal and therefore, have limited ranges of distribution (Sato et al., 2012; Tedersoo et al., 2020). Although the major mycorrhizal partners of terrestrial orchids appear to favor a cross-scale distribution with specificity ranging from wide to very narrow (Jacquemyn et al., 2017; Swarts and Dixon, 2017), comparatively little is known about the diversity and biogeography of the associated mycorrhizae of epiphytic orchids. Therefore, the interaction between OMF and orchids is a key factor determining orchid distribution and development, and diversity of OMF has a strong impact on the niche and life cycle of host orchids. For instance, low OMF diversity and high heterogeneity in OMF community composition may lead to weak growth of orchid populations (Kaur et al., 2020).

Orchids exhibit astonishing morphological characteristics, such as labella and modified petals, that indicate their substantial adaptability to the environment (Zhang et al., 2018). On one hand, interactions with specific pollinators promote the reproduction of orchids; on the other hand, symbiotic fungi are required for soil exploitation (Selosse, 2014). Greater environmental heterogeneity and a wider range of resource availability usually contribute to the increase of orchid species diversity (Schödelbauerová et al., 2009; Traxmandlová et al., 2018). Thus, untangling the role of environmental conditions in determining the distribution and abundance of orchids is a prerequisite for effective conservation of these species. Recently, Djordjević and Tsiftsis (2020) systematically sorted out the effects of environmental factors on the distribution, abundance, and richness of orchids. For instance, rainfall and light regime in the habitat are closely related to the flowering patterns and population dynamics of orchids (Wells and Cox, 1991; Jacquemyn et al., 2010a); physical and chemical properties of soil (such as pH, soil moisture, nutrients, etc.) significantly affect the performance of orchid populations (Stuckey, 1967; Tsiftsis et al., 2012). Interestingly, there is growing evidence supports that coexisting orchid species usually exhibit strongly spatially segregated distribution patterns due to strong clustering within individual species and small overlap between species, and that they are often associated with different OMF communities, which are largely explained by differences in soil moisture and pH (e.g., Jacquemyn et al., 2007, 2012, 2014, 2015a; Waud et al., 2017; Chen et al., 2019b; Kaur et al., 2019). These clues strongly suggest that environmental factors may indirectly affect orchid distribution and population dynamics by driving niche partitioning in OMF communities. Similarly, OMF can decompose carbon and nitrogen sources in soil organic matter and transfer them to the associated host orchids (Rasmussen, 1995). In addition, orchids often share EcM with neighboring trees, hence orchid mycorrhiza may mediate the significant effect of vegetation types on orchid niche partitioning (Waterman and Bidartondo, 2008; Jacquemyn et al., 2016a).

Orchid fungi are divided into OMF and orchid non-mycorrhizal fungi (ONF) based on whether or not functional pelotons are present in cortical cells. OMF include at least 17 families of basidiomycetes and five families or genera of ascomycetes (Dearnaley, 2007; Dearnaley et al., 2012). Tulasnellaceae, Ceratobasidiaceae, and Serendipitaceae are most commonly known as rhizoctonia-type Basidiomycetes. Basidiomycetes and Ascomycetes are relatively abundant in the tree roots of forest ecosystems and cultivated species worldwide (Cregger et al., 2018; Wang et al., 2019a; Trivedi et al., 2020), and are commonly associated with orchids as well. Interestingly, a large proportion of ascomycetes associated with orchids are ONF. For example, Helotiales endophytes are the dominant group of ONF associated with host orchids in different habitats as well as major players in plant–fungus associations in a variety of forest ecotypes (Toju et al., 2013; Jacquemyn et al., 2016a, 2017). Previous studies have identified more than 110 genera of ONF, including 76 genera of ascomycetes and 32 genera of basidiomycetes (Ma et al., 2015). Although ONF are often overlooked, recent studies have highlighted their importance in the promotion of orchid seed germination and performance as well as changes in the composition of key chemicals such as sugars. Moreover, they also play a role in mobilizing soil nutrients in the rhizosphere to improve orchid viability and environmental adaptability, and serve as new sources of phytochemicals and bioactive substances to protect the host from soil pathogens. Hence, these ONF provide the plants with promising medicinal and agricultural breeding prospects (Chen et al., 2006; Yuan et al., 2009; Aly et al., 2010; Novotná et al., 2018; Sisti et al., 2019; Hajong and Kapoor, 2020; Wu et al., 2020). Furthermore, ONF may interact with OMF to influence the distribution and population dynamics of orchids. Therefore, the diversity and functionality of ONF should be further explored to gain insight into the associations between orchids and fungi as a whole. In addition, studying how ONF and OMF make rational use of the ecological niches in orchid rhizospheres (i.e., their coexistence mechanism) and the correlation between their distribution and the phylogenetic eigenvectors of orchid species could help us better understand orchid mycorrhizal ecology. We believe that the development of molecular methods and genomics techniques enables the simultaneous consideration of both OMF and ONF in mycorrhizal ecology. This may breed a more complete and informative fungal network that can more accurately predict their association with environmental variables, which may be a key to igniting a new wave of research into orchid mycorrhizae.

**Figure 1:**
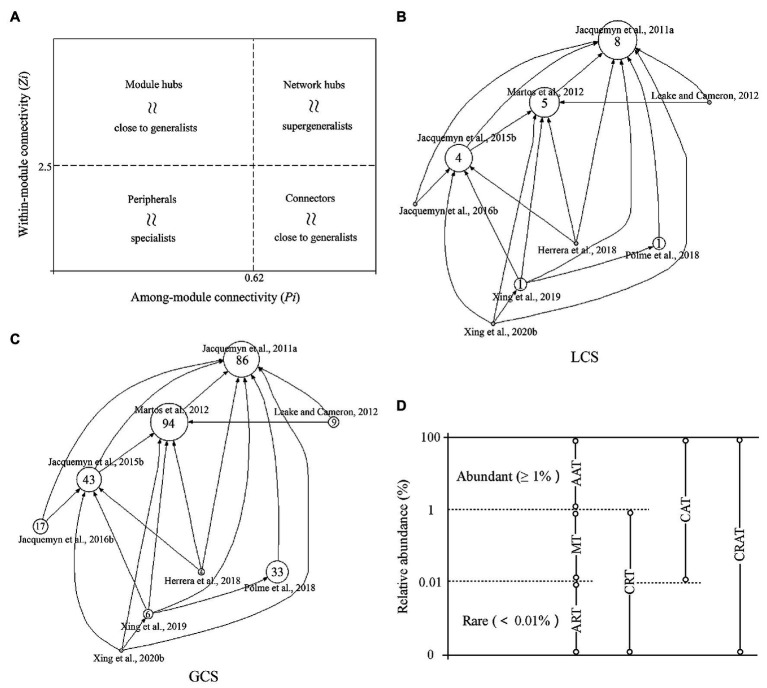
**(A)** *z*–*P* plot depicting the role of each species in the interaction network. Peripherals might represent specialists whereas module hubs and connectors are close to generalists and network hubs are supergeneralists. **(B,C)** Local citation score (LCS) and global citation score (GCS) citation diagrams of all orchid mycorrhizal network research papers retrieved from the core Database of Web of Science. Each circle represents a research article. The numbers in the circle are the LCS **(B)** and GCS **(C)** of the corresponding literature, and the size of the circle is proportional to citation scores. Arrows indicate cross-references between the studies. **(D)** Definition and classification of microbial taxa: (i) Always abundant taxa (AAT), operational taxonomic units (OTUs) with abundance always ≥1% in all samples; (ii) Always rare taxa (ART), OTUs with abundance always <0.01% in all samples; (iii) Moderate taxa (MT), OTUs with abundance between 0.01 and 1% in all samples; (iv) Conditionally rare taxa (CRT), OTUs with abundance <0.01% in some samples and below 1% in all samples; (v) Conditionally abundant taxa (CAT), OTUs with abundance greater than 0.01% in all samples and ≥1% in some samples but never rare (<0.01%); and (vi) Conditionally rare and abundant taxa (CRAT), OTUs with abundance varying from rare (≤0.01%) to abundant (≥1%).

The thousands of complex and highly dynamic interactions between plants, microbiomes, and the environment can be analyzed using ecological networks that play pivotal roles in associations between OMF, EcM, and arbuscular mycorrhizae (AM) fungi, and are often used as black boxes to study the transfer of carbon signals across hosts (Banerjee et al., 2018; Tedersoo et al., 2020). Modular analysis within these networks can be used to identify key microbial groups that are closely related to plant growth and yield. Additionally, the topological roles of the species included in complex networks can be simplified into four categories based on within-module connectivity (*Zi*) and among-module connectivity (*Pi*; Figure 1A). Connectors, module hubs, and network hubs are considered ecosystem engineers, which have significant impact on the assembly of communities and can support higher levels of ecosystem function. Network hubs are further defined as keystone taxa due to their high connectivity within these networks, and have more important functional attributes and high interpretation rates on the dynamics of plant microorganisms (Deng et al., 2012; Cavieres et al., 2014; Banerjee et al., 2018; Ma et al., 2020). Thus, they can be used to manipulate the function of the microbiome or predict changes in its community composition. A query of the Web of Science core database using the keyword “orchid mycorrhizal network” retrieved a total of nine articles (as of 15 October 2020), focusing mainly on the interaction between epiphytic orchids in tropical systems or terrestrial orchids in temperate systems and OMF. The methods, ecological premise and revealed network architecture in the retrieved articles are described in Supplementary Table S1. We used the HistCite software to analyze the citation network and calculate the local citation score (LCS) and global citation score (GCS) from the retrieved articles (Figures 1B,C). In general, research on the mycorrhizal network of orchids appears to be insufficient, greatly restricting our understanding of the stability and persistence of the species-rich orchid community.

Hence, in this study, we summarize the effects of OMF on orchid distribution and population dynamics, elucidate OMF networks and the phylogenetic relationships in orchid–OMF interactions. More specifically, we would like to discuss the progress in these three aspects to preliminarily clarify the following two issues: (1) the mutual selection mechanism of orchid–OMF and the role of ecology and evolution in this pattern? and (2) the relative importance of nestedness and modularity in orchid mycorrhizal networks? Finally, in the “Prospects” section, we focus on the functional roles of rare groups and discuss several key issues in symbiosis that could benefit from the advancement of OMF network research. Collectively, we summarize the results or conclusions of some case studies related to the target topic, and attempt to focus on the emerging patterns that are more consistent in these summaries. However, it should be noted that the network perpective applies mainly to tropical orchids, and more datasets are needed to expand other biomes. Through this review, we hope to direct attention toward the insufficiently explored field of orchid–fungus mutualism, and encourage the use of beneficial fungal groups for protection of endangered orchids.

## Effects of OMF on Orchid Distribution and Population Dynamics

The distribution and abundance of orchid populations are curtailed by biotic and abiotic factors. These include latitude, macroclimate, area size, and evolutionary history at the large landscape scale, soil characteristics, light conditions, substrate types, degree of disturbance, pollinating insects, and seed production and dispersal at the local landscape scale, while the effects of altitude, soil moisture, and pH may span both scales (McCormick and Jacquemyn, 2014; Djordjević et al., 2016; Traxmandlová et al., 2018; Tsiftsis et al., 2019; Djordjević and Tsiftsis, 2020). The influence of these factors may depend on the spatiotemporal scale in consideration. Although orchids have dust-like seeds that generally lack nutrients, OMF play a critical role in seed dispersal and germination, establishment of new seedlings, and soil niche partitioning of their lifelong host orchid (McCormick et al., 2006, 2018; Selosse, 2014; Tedersoo et al., 2020). In natural habitats, microhabitats rich in OMF generally facilitate more seed germination and seedling establishment, and more orchids tend to grow in areas with richer distribution of OMF. As a result, changes in OMF composition, abundance, and evenness may greatly influence the fitness of orchids, which in turn affects the distribution and community composition of orchids (McCormick and Jacquemyn, 2014; Waud et al., 2016a; McCormick et al., 2018).

## Effects of OMF Abundance on Orchid Distribution

To our knowledge, no reports show that the distribution of orchids at large scales is restricted by OMF. While Hemrová et al. (2019) suggested that fungal symbionts are the main driving forces for the distribution of forest orchids at the landscape scale based on germination experiments and species distribution models integrating multiple habitat characteristics, they did not find molecular evidence for OMF diversity. A possible explanation for this is that OMF has a wide biogeographic distribution, and some major branches have been witnessed on a broad landscape scale (Jacquemyn et al., 2017). Hence, the distribution of OMF assemblages *per se* may not be a limiting factor for the distribution of orchids. However, extensive studies sampling orchids across continents would be required to determine whether the total fungal community enlarges or reduces the distribution of host orchids on the landscape scale. Studies on the local restriction of orchid distribution by OMF abundance include at least nine reports covering 13 specialized orchids (reviewed in McCormick et al., 2018). Most results demonstrate that the germination percentage of seeds is higher near adult orchids where OMF abundance tends to be greater. In addition, some studies show significant positive correlations between mycoheterotrophic orchid abundance and OMF abundance, strongly suggesting that OMF affects the abundance of orchids (McCormick et al., 2009). However, current evidence showing the influence of OMF abundance on the distribution of orchids is mainly based on seed germination and protocorm development (McCormick et al., 2012, 2016, 2018; McCormick and Jacquemyn, 2014). Subsequent studies on the later developmental stages of orchids might unravel more information.

Most orchids experience carbon restrictions during nutrient dormancy. During this phase, orchids require fungi to provide nutrients, and the recovery from dormancy is proposed to be linked to the local abundance of appropriate OMF (Rock-Blake et al., 2017; McCormick et al., 2018; Shefferson et al., 2018). Therefore, OMF abundance may affect the apparent density of orchid populations by affecting the dormancy process, thereby affecting the population characteristics and reproductive success. At the same time, OMF inoculation experiments revealed that the introduction of OMF in areas without orchids but near existing orchid populations can be successful, so that OMF abundance may also increase seed germination and the protocorm formation rate (McCormick et al., 2012, 2016). Interestingly, the relationship between the OMF abundance and the distribution of orchids suggests that the location of adult orchids determines the richness and abundance of OMF to some extent. In addition, several reports demonstrate that fungi obtain nutrients from autotrophic orchids (Cameron et al., 2006; Hynson et al., 2009; Liebel et al., 2015; Yeh et al., 2019). Therefore, we cannot rule out the possibility that the abundance of OMFs may be caused by the density of autotrophic orchids. Furthermore, it is essential to design more tests to explore factors controlling the abundance of OMF and their impact on individuals and populations of orchids.

## Effects of OMF Identity and Spatiotemporal Variability on Orchid Distribution and Population Dynamics

Most orchids are combined with different OMF in different habitats or climatic conditions, indicating that beneficial OMFs can vary across these conditions (Martos et al., 2012; Jacquemyn et al., 2016a,b; Duffy et al., 2019; Xing et al., 2020a). Germination and growth promotion tests have largely shown that OMF exhibit some specificity (e.g., Zi et al., 2014; Rasmussen et al., 2015; Meng et al., 2019; Zhang et al., 2020), while some studies have suggested that only a few members of OMF assemblages can promote the growth and flowering of orchids under various environmental stresses (McCormick et al., 2009, 2018). Therefore, the identities of OMF and their interactions with the environment may make certain orchid–OMF assemblages more beneficial to the growth of orchids, positively affecting their distribution and population dynamics. In addition, Nurfadilah et al. (2013) found that fungi associated with some widely distributed Australian orchids were more likely to acquire nutritional resources than fungi associated with orchids found only in certain habitats. This has also been confirmed at the genotype level by subsequent multi-omics studies (Kohler et al., 2015; Fochi et al., 2017a,b). Interestingly, *Tulasnella* and some *Serendipita* fungi lack genes for using nitrate and nitrite, though these genes are commonly found in *Ceratobasidium*. Moreover, these three types of rhizoctonias possess different genes that help in the absorption of carbon substrates. Accordingly, recent tests on the sensitivity of orchid seed to nitrate concentration have revealed that nitrates can affect the distribution of orchids by directly inhibiting seed germination (Figura et al., 2020). Hence, nitrates have inhibitory effects on seed germination, growth, and persistence of orchids. With the change of landscape, the rapid increase in nitrate content in intensive pastures or meadows inhabited by a large number of orchid species due to the high soil nitrification rate and the increasing atmospheric nitrogen deposition around the world (Figura et al., 2020; Moore et al., 2020), and eutrophication of habitats (particularly in habitats with severe human disturbance, such as some ancient tea estates in southwest China, the rich orchid resources are faced with the impact of heavy use of chemical fertilizers and human destruction) poses a potential threat to the distribution of orchids. It is worth mentioning that a recent study corroborated that some *Ceratobasidium* symbionts can effectively alleviate the inhibitory effect of nitrate on orchid seed germination (Figura et al., 2021). In addition, recent evidence shows that some cyanobacteria species with nitrogen-fixing activities are present in the velamen of epiphytic orchids (Deepthi and Ray, 2020). Therefore, in the epiphytic niches, *Tulasnella* and some *Serendipita* fungi that cannot use mineral nitrogen may benefit from the velamen roots of epiphytic orchids, at least during adulthood; indicating that the notion that OMF are free-living fungi may need to be reviewed, because epiphytic orchids may indirectly affect OMF performance by recruiting cyanobacteria species.

In addition to the distribution, abundance, and identity of OMF, spatiotemporal variation in OMF may more important factor in determining the distribution and population dynamics of orchids. The high spatiotemporal turnover rate of OMF may also reduce competition for resources, *via* niche separation, by promoting the coexistence of more orchid species in the natural environment. This is corroborated by several studies on the coexistence of terrestrial orchids that use a combination of spatial point pattern analysis and OMF phylogenetic analysis. These studies have revealed that coexisting orchid species have different OMF communities with little overlap over fine spatial scales, and that individual orchid species commonly occur as high-density clusters, displaying high local dominance (Jacquemyn et al., 2012, 2014, 2015a; Waud et al., 2016b). Therefore, the co-occurrence of terrestrial orchids observed in nature may be mediated by spatial distribution and interactions of the associated OMF. However, minimal research has been conducted on the relationship between the spatial distribution of epiphytic orchids on different phorophytes and OMF. Since epiphytic orchids account for about 70% of orchids (Dearnaley et al., 2012; Chase et al., 2015), the lack of such research limits our understanding of how OMF affect the distribution and population dynamics of orchids.

## Roles Of *Tulasnella* in Orchid Community Construction

The fungi *Tulasnella* mostly reside in various orchid tissues (mainly roots and stems) and tend to be extremely sensitive to environmental variables, indicating a strong dependence on host orchids or surrounding plants. Therefore, recovery from disruption of the multi-nutrient balance among *Tulasnella*, orchids, and accompanying plants (the plant species that often occur around a certain orchid species and do not belong to the Orchidaceae in natural habitats and are expected to have a specific association with the orchid species) that is established by long-term coevolution could be difficult. This can result in orchid breeders or orchid enthusiasts facing problems, while reestablishing conditions for growing orchids. Studies have shown that the use of peat-based, a mixture of coconut shells, bark shavings, soil from the original habitat, or a mixture of pinecone scales, mosses, and humus, can induce symbiosis between transplanted orchid individuals or asymbiotically cultured orchid individuals grown *ex situ* and Tulasnellaceae fungi (Han et al., 2016; Kaur et al., 2018; Qin et al., 2019). However, no *Tulasnella* symbiotic with orchid individuals were obtained when transplanted in original habitat soil or sawdust (May et al., 2020; Li et al., unpublished data). Preliminary research suggests that the dominant OMF and ONF in fungal communities reconstructed by cultured orchid individuals are different from those seen in wild populations. Overall, fungi belonging to the groups Atractiellales, Auriculariales, Ceratobasidiaceae, and *Fusarium* tended to increase, while the abundance of Tulasnellaceae and Pyronemataceae tended to decrease (Downing et al., 2017; Qin et al., 2019; May et al., 2020), as a consequence of transplantation, and the extent of such changes may depend on the time scales of culture or transplantation. This indicates low survival rates of cultured orchids, which may be caused by the loss of some key OMF (e.g., Tulasnellaceae fungi) or the restricted construction of new mycorrhizal communities. Hence, selecting substrates with high OMF diversity, using molecular identification of OMF composition of substrates, is critical for improving culturing practices. However, further research is required to determine the extent of similarity in *Tulasnella* taxa between cultivated and wild orchids across different time scales.

## A Framework for how OMF Affect the Distribution and Population Dynamics of Orchids

Orchid mycorrhizal fungi exhibit widespread biogeographical distributions with major clades found all over the world, suggesting that the widespread distribution of orchids is driven by OMF (Jacquemyn et al., 2017). However, the mechanisms by which OMF affect the distribution and population dynamics of orchids are still poorly understood since most current knowledge is based on molecular data from adult plant symbionts, while complex mycorrhizal associations with orchids occur at different stages of their life cycle. In this paper, we postulate a simple framework for the effects of OMF on orchid distribution and population dynamics, though this conception may be biased. We argue that orchid–OMF associations exhibit complementary and specific effects of selection that are highly adapted to the environment, and promote the niche breadth of orchid species, which may act as a stabilizing force. More specifically, orchid species with specific mycorrhizae are usually symbiotic with generalist OMF, while OMF associated with host orchids with generalist mycorrhizae are often limited in their distribution. In other words, the distribution of orchids is shaped by coupled influences of environmental variables and efficient complementary selection between OMF and orchids. The most well-known example of association between generalist OMF and orchid species with mycorrhizal specificity is that of Serendipitaceae, which are symbiotic partners specifically associated with many host orchids. Serendipitaceae are also widely distributed, shared by orchids and their accompanying plants in several habitats, or serving as a beneficial growth-promoting fungus for a wide range of agricultural crops (e.g., Davis et al., 2015; Jacquemyn et al., 2015a; Fritsche et al., 2020; Reiter et al., 2020). While *Platanthera leucophaea*, which is protected by the United States federal government, is highly dependent on *Ceratobasidium* in the tallgrass prairie ecosystems of North America, *Ceratobasidium* species has also been isolated from various orchid species found in other locations (Thixton et al., 2020). Similarly, two rare *Orchis* sister species have high specificity for the dominant fungal symbiont *Tulasnella helicospora*, even though this fungus is found across the world (Calevo et al., 2020). Contrarily, generalist mycorrhizal orchids recruit a large number of symbiont partners, but these compatible OMF are rarely found in other areas (Jacquemyn et al., 2011a). While biogeography is the main factor affecting the microbial communities (including fungi and bacteria) associated with *Gymnadenia conopsea* (i.e., composition varies greatly with location), the species still exhibits certain specificities (Lin et al., 2020; Xing et al., 2020a). Similarly, Gao et al. (2020) found that OMF isolated from orchid species that coexist with *G. conopsea* in the wild do not promote its seed germination and protocorm formation *in vitro*, since they require specific OMF.

Environmental filtering largely accounts for the narrow distribution of OMF associated with these generalist mycorrhizal orchids. Recent evidence indicates that phosphorus content is higher in the roots of larger populations of *Platanthera cooperi* and the surrounding bulk soil, which are mainly colonized by the Tulasnellaceae. In contrast, higher zinc content and a higher relative abundance of Ceratobasidiaceae are observed in smaller *P. cooperi* populations (Kaur et al., 2020). Interestingly, Ceratobasidiaceae are more abundant in phosphorus-rich restored grasslands, while Serendipitaceae are more common in semi-natural grasslands with higher organic matter content (Vogt-Schilb et al., 2020). This may be due to differences in how variables in a particular habitat are weighed. In addition, it has been widely reported that several environmental variables (such as soil water content, pH, and soil nitrogen content) can affect the composition and abundance of OMF communities (Jacquemyn et al., 2015a; Waud et al., 2017; Duffy et al., 2019; Kaur et al., 2019; Mujica et al., 2020). Hence, the structure of OMF communities is significantly related to microenvironmental changes. Thus, these factors exert a joint effect on the formation and structure of orchid populations.

It must be noted that this framework applies mainly to terrestrial orchids. Since orchid–OMF interactions are of a higher order due to the presence of abundant phorophytes in epiphytic orchids, equilibrium dynamics underlying their mutual selection are complicated. More scenarios need to be considered to address this problem though a recent study has shown that phorophytes and epiphytic orchids harbor different fungal communities (Eskov et al., 2020). Moreover, there should be greater focus on the fungal taxa associated with epiphytic orchids, epiphytic niches, and accompanying plants as well as their mutual selection mechanisms. Lastly, the orchid–OMF complementary selection mechanisms may be related to evolutionary constraints, which will be discussed in detail in the following section.

In summary, the patchy distribution, heterogeneous abundance, identities, and spatiotemporal variability of OMF have crucial effects on the local distribution and population dynamics of orchids. The local distribution of orchids may in turn promote the formation and diversification of orchid species by curtailing the population size and gene flow among populations, which may be responsible for the huge species diversity of Orchidaceae. Since the distribution of orchids is affected by various factors, the relationship between OMF and the distribution of orchids should be further explored. Future studies must particularly focus on the influence of availability of OMF, flow of nutrient resources between orchids and OMF, and abiotic factors on the distribution of tropical orchids.

## Phylogenetic Signals in Orchid–OMF Interactions

The composition and distribution of biological assemblages are strongly influenced by a series of ecological and evolutionary processes (Heilmann-Clausen et al., 2016; Boeraeve et al., 2018; Beng and Corlett, 2019; Wang et al., 2019a). The genetic relationship between hosts in antagonistic or mutualistic interactions and its influence on the assembly of fungal communities has been unraveled by recent studies (e.g., Põlme et al., 2013; van der Linde et al., 2018). For example, different degrees of evolutionary constraints have been observed in AM, EcM, plant pathogens, and fungi in general (Erlandson et al., 2018; Wang et al., 2019a,b; Yang et al., 2019). In addition to these biotrophic fungal guilds, the dissimilarities among free-living soil fungal communities significantly increase over large spatial scales and with increasing plant phylogenetic distance, however, the explained variation is relatively lower than that of pathogens and EcM fungi (Yang et al., 2019). Controlled experiments also suggest that soil microorganisms that are obligately symbiotic with some trees usually promote growth on these trees or closely related species, but do not affect growth as much on distantly related species (Liang et al., 2019). These results suggest that closely related hosts usually have certain fungal specificities. Considering the dependence and specificity of orchids on OMF, the evolution of host orchids may be a key factor affecting the composition of the OMF community.

In recent years, some studies have indicated the existence of phylogenetic conservatism in the interplay between orchids and OMF. Jacquemyn et al. (2011b) revealed that the phylogenetic structure of 16 species of the genus *Orchis* distributed across 11 different regions in Europe can explain the community differences in associated Tulasnellaceae. Many orchid species that are closely related within genera host similar rhizoctonias or Tulasnellaceae operational taxonomic units (OTUs), such as *Cypripedium* (Shefferson et al., 2007), *Goodyera* (Shefferson et al., 2010), *Neottia* (Těšitelová et al., 2015), *Teagueia* (Suárez and Kottke, 2016), *Caladenia* (Phillips et al., 2016), *Dendrobium* (Xing et al., 2017), *Pleione* (Qin et al., 2019), and Cypripedioideae (lady’s slipper subfamily; Shefferson et al., 2019). Therefore, orchid–OMF specific associations during their evolutionary history may result in strong influences of orchid phylogeny on OMF communities. However, rhizoctonias display little or no coevolution with host orchids. This indicates asymmetric interactions during coevolution process and phylogenetic conservatism of functional traits of orchids (De Deyn and van der Putten, 2005; Heilmann-Clausen et al., 2016; Wang et al., 2019a).

Nevertheless, studies on the interactions between orchids of multiple genera and rhizoctonias have produced inconsistent conclusions. Martos et al. (2012) performed a phylogenetic analysis of tropical orchids and symbiotic rhizoctonias distributed on Reunion Island, utilizing a narrow resolution (34 angraecoid species) and a broader resolution (25 orchid genera), and showed that the overall phylogenetic signal was weak. At a narrow resolution, the evolutionary constraints between orchids and rhizoctonias tended to be asymmetric, with phylogenetic signals only observed in orchids. Interestingly, at the broader resolution, both orchids and rhizoctonias in the epiphytic sub-networks displayed significant phylogenetic signals. Similarly, a recent analysis of the mycorrhizal association of 44 tropical orchids covering three life forms (terrestrial, epiphytic, and lithophytic) with rhizoctonias or *Tulasnella*, revealed low phylogenetic signals in both orchids and fungi (Xing et al., 2019). Moreover, no significant differences were observed in phylogenetic signals between the three types of orchids and the sub-networks formed by rhizoctonias or *Tulasnella* separately, which were close to zero. Differences in the results of these two studies could be related to the phylogenetic spectra of the orchids involved (Tedersoo et al., 2014; Xing et al., 2019). The number of terrestrial, epiphytic, and lithophytic orchid species in the latter study was less than 20, and the phylogenetic diversity focused on fewer orchid genera. No significant phylogenetic signals were consistently detected on either side of the interactions in the binary network formed by seven species of orchids and rhizoctonias belonging to different genera distributed in Song Mountain, Beijing (Chen et al., 2019b). Thus, further research could sample greater numbers of different types of orchids focusing on the broader orchid phylogenetic spectra. Furthermore, the associations of phylogenetically related host orchids with similar fungal communities in rhizosphere soil and orchid-occupied bulk soil may be worth investigating.

The phylogenetic niche conservatism theory proposes that host species with close genetic relationships tend to possess highly similar morphologies and functions (Losos, 2008). There is substantial evidence for the effects of phylogenetic eigenvectors and species-specific functional traits on fungal communities often overlap significantly. The phylogenetic effects of hosts can be explained by the conservatism of plant functional traits. In addition, the phylogenetic relatedness of hosts could explain the similarity of functional traits to a large extent, which would allow a rough prediction of either of these features based on the other (Wardle et al., 2004; Legay et al., 2014; López-García et al., 2017; Wang et al., 2019a; Yang et al., 2019). This is probably one of the major reasons for the greater likelihood of observing phylogenetic signals from the same orchid genus during interactions with rhizoctonias. However, since data regarding root traits that are important in the construction of underground communities is lacking, further investigations are required to examine the extent to which evolutionary constraints of orchid genera are caused by their own functional traits. In addition, little to no phylogenetic signals were observed in the narrow phylogenetic spectra of orchids. This could be because the associated rhizoctonias were mostly saprotrophic and endophytic fungi (Smith and Read, 2008; Jacquemyn et al., 2017) with relatively high functional redundancy (particularly saprotrophic fungi) and sensitivity of local species pools to abiotic environmental filtering, which would substantially obscure the influence of orchid phylogeny (Setälä and McLean, 2004; Erlandson et al., 2018).

When a plant invades or is transplanted into a new environment, existing microorganisms in the environment may adapt or be redistributed. Over time, the outcomes of these adaptations may not be very beneficial to hosts of other genotypes (Batstone et al., 2020), which indicates that shared evolutionary history is an important factor in the mutual selection between hosts and microorganisms. Consistent with this view, recent studies have shown that “invasive orchids were capable of associating with a broader range of mycorrhizal fungi than co-occurring native congeners (a generalist strategy)” but they were also less likely to harbor pathogenic fungal groups (Downing et al., 2020). However, continual monitoring on longer time scales is required to verify whether beneficial associations developed by invasive orchids are driven by evolutionary history.

Hence, to summarize these two sections, the composition of OMF communities are determined jointly by ecological and evolutionary constraints, the relative importance of which depends on the specific time, space, and orchid species studied. Subsequent case studies could determine the contributions of these constraints through variation partitioning analysis (VPA) and help us understand the relationship between orchid ecology and evolution.

## Orchid Mycorrhizal Network

The architecture of plant–fungus interactions varies according to the mycorrhizal type. The association network of AM and plants is usually characterized by a nested assembly such that host plants that are symbiotic with fewer AM prefer to form symbiotic associations with AM that are symbiotic with most host plants (Chagnon et al., 2012; Montesinos-Navarro et al., 2012). Orchid mycorrhizae and ericoid mycorrhizae (ErM) interaction networks form a modular structure (Martos et al., 2012; Jacquemyn et al., 2015b; Toju et al., 2016; Xing et al., 2019) with high specificity between host plants and partners, while EcM network architectures tend to assume an intermediate structure (Bahram et al., 2014; van der Heijden et al., 2015). Recently, Põlme et al. (2018) performed a meta-analysis of 111 datasets of plant–fungus interactions, which showed that the OMF community responded most strongly to orchid host identity, with significantly higher levels of specificity than other types and higher modularity than EcM and AM. In general, the orchid mycorrhizal network has significant characteristics of modules as a whole. However, the contributions of modularity and nestedness in the local network often change, and the orchid–OMF interaction in different ecosystems shows inconsistency with the whole in network eigenvalues. Temperate and Mediterranean ecosystems exhibit slightly different architectures: the former tends to be significantly nested (Jacquemyn et al., 2011a, 2015b, 2016b), whereas tropical orchids and OMF symbiosis are more diversified (Martos et al., 2012; Kottke et al., 2013; Herrera et al., 2018; Xing et al., 2019, 2020b).

The distribution of orchid species and OMF and their selective effects constitute a complex orchid mycorrhizal network, and the tight junctions present in the network are particularly important for the coexistence and population dynamics of orchid species. Jacquemyn et al. (2011a) applied network analysis for studying symbiotic relationships between orchids and OMF for the first time, and analyzed the architecture of the interaction between 16 *Orchis* species distributed in 11 regions of Europe and OMF. From this study, they confirmed that the interaction between orchids and OMF at the community level showed a nested structure similar to a mutualistic relationship network seen in pollination and seed dispersal, as well as networks of predation. This study was identified as a pioneering work in the field of orchid mycorrhizal networks based on our LCS analysis (Figure 1B). To our knowledge, this is also one of the first studies to apply network analysis methods to provide insight into complex mycorrhizal symbiotic relationships. Immediately afterward, Martos et al. (2012) built a binary network of nearly half of the tropical orchid species and 95 rhizoctonia fungi associated with them on Reunion Island, and found that the overall orchid mycorrhizal network showed high modularity due to the ecological barrier between epiphytic and terrestrial orchids. However, the epiphytic subnetwork formed a highly nested pattern. This study constructed the largest orchid mycorrhizal network to date, which was another important milestone in the progress of the orchid mycorrhizal network and continues to influence fields other than orchid mycorrhizal networks (Figure 1C). Subsequently, three studies further supported the highly modular structure of the orchid mycorrhizal network. The structure of the mycorrhizal network, formed by species of the genus *Dactylorhiza* with different levels of ploidy and inhabiting a wide range of habitats (including acid peat bogs, wet alkaline grasslands, dry meadows, and forests), is characterized by modularity that is significantly dependent on local environmental conditions (Jacquemyn et al., 2016b). Although orchid species of different life forms are all simultaneously symbiotic with multiple OMF, the overall interconnected network remains highly modular due to the enhanced specificity of Tulasnellaceae from terrestrial to epiphytic or lithophytic orchids (Xing et al., 2019). Interestingly, overall OMF diversity in a narrow transect of 10 × 1,000 m with relatively similar habitats could be partitioned into a subset of 20 terrestrial orchids with mycorrhizal diversity belonging to five coexisting genera, low overlap among the subsets, and multiple isolated groups present in the interconnected network (Jacquemyn et al., 2015b).

In contrast, four studies supported significantly nested network features. The nested network formed between the highly diverse epiphytic orchids and rhizoctonia distributed in tropical montane rainforests of southern Ecuador may be influenced by climate, as climate is the main driving force for OMF community turnover among sites (Kottke et al., 2013). Herrera et al. (2018) further confirmed that terrestrial and epiphytic orchids shared abundant Tulasnellaceae mycobionts in different habitats within tropical forests of southern Ecuador, and the network showed a nested structure with generalists forming the core. Due to the large degree of overlap seen in the mycorrhizal communities of epiphytic and lithophytic orchids, the network structure formed by these two types of orchids and sympatrically distributed terrestrial orchids is highly modular but also shows significant nestedness (Xing et al., 2019; Qin et al., 2020). Notably, the OMF network of *Dendrobium* species inhabiting the same phorophyte escaped strong selection by the host and showed significant asymmetric specialization (Xing et al., 2020b). In addition, consistent with pollination networks, robustness analysis revealed that generalist OMF and orchid species play an important role in the stability of interrelated networks, and their loss may drive the cascading loss of biodiversity (Memmott et al., 2004; Burgos et al., 2007; Herrera et al., 2018). Moreover, the robustness of the symbiotic network formed by terrestrial and epiphytic orchids was only slightly different, implying that OMF is equally important for the fate of both life forms of orchids (Herrera et al., 2018).

## Formation of Significantly Nested Structures

Although there was no significant difference in community nestedness among different mycorrhizal types, it was significantly negatively correlated with annual average rainfall. Moreover, the nestedness values of orchid mycorrhizal networks showed large variation, implying that the significant nested structures formed by orchids and OMF may be more sensitive to fluctuations in environmental conditions (Põlme et al., 2018). This is consistent with the hypothesis proposed by Kottke et al. (2013) that climate may be the cause of nested networks. More specifically, the similarity of OMF communities resulting from habitat variation may explain the observed nested structure, such as the significant nested structure seen in *Orchis* species due to low variation in habitats. Contrarily, *Dactylorhiza* species exhibit greater habitat differentiation, resulting in rare overlap among OMF communities between different populations and a highly modular structure (Jacquemyn et al., 2011a, 2016b). From an evolutionary point of view, mycorrhizal associations of some species of *Orchis* and *Cypripedium* may be undergoing expansion of phylogenetic breadth, presenting broad specificity, and gradually driving network attributes to have generalists at the core order to maximize adaptation to the environment and absorb nutrients (Shefferson et al., 2007; Jacquemyn et al., 2011b).

In addition, the research scale and the threshold for species delimitation may be two important factors affecting network nestedness. The smaller the scale of the network constructed, the tighter interaction within the network, resulting in increased nestedness of the network (Caruso et al., 2012; Öpik and Moora, 2012; Chagnon et al., 2016). Thus, sampling should be performed at the similar scales to compare the mycorrhizal network of coexisting orchid species in different habitats. As the OTU sequence similarity threshold increases, the number of OTUs and rare associations increase, while the strength of nestedness decreases (Toju et al., 2014; Põlme et al., 2018). However, the pattern of nestedness observed in the orchid mycorrhizal network did not vary according to the OTU delimitation threshold, probably because most of the OMF considered in these studies were highly abundant species (Jacquemyn et al., 2011a; Herrera et al., 2018). Considering the importance of rare species in subsurface ecosystem services, the impact of OTU classification on network nestedness should be fully considered, while performing in-depth analysis of orchid–fungal (including ONF) networks.

## Formation of Highly Modular Features

Since coexisting orchid species commonly exhibit highly spatially clustered and strongly spatially segregated distribution patterns and are often associated with different OMF communities, and these OMF communities with patchy distributions rarely overlap (Jacquemyn et al., 2007, 2014; Waterman et al., 2011; Waud et al., 2016b), the interaction network between orchids and OMF often shows highly modular characteristics. On the one hand, this may be due to the specific selection of OMF taxa by host orchids (Jacquemyn et al., 2015b; Xing et al., 2019). In order to maximize mutual symbiosis in a complex environment, hosts usually allocate more carbohydrates to better quality partners, resulting in increased levels of specificity for the association between orchids and OMF, which in turn form a symmetrical and modular network structure of interactions (Kiers et al., 2011). Notably, the host selection effect of ErM associations is low but still presents a high degree of modularity, which may be explained by the high sensitivity of the modularity metric to total links in the dataset (Bahram et al., 2014). Moreover, the modularity of networks may be due to environmental variables that enhance specific selection of OMF by hosts (Jacquemyn et al., 2010b; Shefferson et al., 2019). Specific environmental gradients or distinct niche differentiation may allow host orchids to be specifically associated with OMF characterized by greater taxonomic richness or functional diversity. In addition, the presence of forbidden links may explain the strong modular structure (Olesen et al., 2011). For example, the inconsistency of spatiotemporal dynamic changes in OMF limits certain pairwise interactions that may occur throughout the network.

## A Framework for Weighing the Relative Importance of Nestedness and Modularity

**Figure 2:**
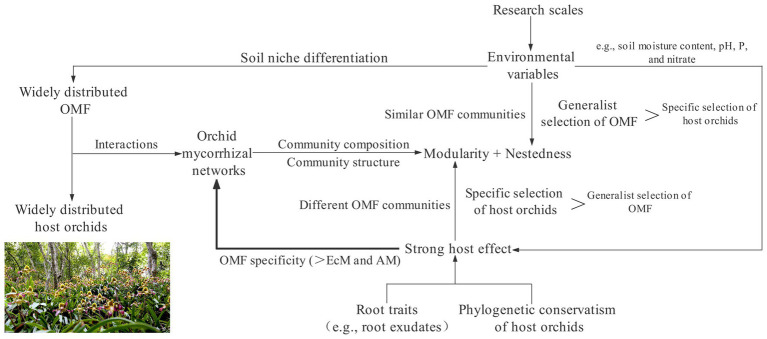
A framework of the architecture of the orchid mycorrhizal network. The intensity between the specific selection of host orchids and the generalist selection of orchid mycorrhizal fungi (OMF) indirectly determines the relative importance of modularity and nestedness by affecting the degree of similarity of OMF communities. The thick line implies that the phylogenetic conservatism and root traits of host orchids as well as environmental variables jointly drive OMF to be significantly more specific than ectomycorrhizae (EcM) and arbuscular mycorrhizae (AM) fungi.

Based on these factors that affect the characteristics of the orchid mycorrhizal network, we initially proposed a framework to weigh the relative importance of nestedness and modularity of the orchid mycorrhizal network (Figure 2). The characteristics of complex mycorrhizal networks formed by orchids and widely distributed OMF mainly depend on the relative strength between specific selection of host orchids and generalist selection of OMF. When the coupled influences of phylogenetic spectra, root traits, and environmental differences of orchids increases the intensity of specific selection of host orchids beyond that by OMF, the network structure is highly modular. Contrarily, when the similarity of environmental conditions drives coexisting orchid species to share similar OMF communities, resulting in greater intensity of generalist selection of OMF, the network structure shows significant nested assembly.

## Future Directions

Co-occurrence network analysis is a promising method to gain insight into ecological communities and may serve as a potential approach to more efficiently and conveniently study the distributions of microorganisms, the pattern of symbiotic relationships, and their impact on plant distribution and population dynamics at the community level. However, this method has not been fully applied to multiple mutualisms of orchids, fungi, and their accompanying plants or phorophytes. Therefore, more attention should be paid to the in-depth analysis of the total fungal co-occurrence network among different habitats (populations) of the same species, different orchid species coexisting in the same habitat, and orchid species of different life forms or at different developmental stages in further studies. In order to increase the accuracy of orchid fungal networks, spatial autocorrelations should be avoided as far as possible when collecting samples and, combinations of analytical methods should be used. Moreover, analytical methods such as IDEN should be developed for the investigation of orchid traits and examining bipartite networks of orchid–fungus interaction, which can help understand cross-kingdom associations between vegetation data and microorganisms (Feng et al., 2019). Alternatively, interactions among orchid-associated fungi can be jointly analyzed by MENA and SparCC, using CoNet, which comprehensively considers multiple correlations, or SPIEC-EASI, which uses a more inferential function (Deng et al., 2012; Faust et al., 2012; Friedman and Alm, 2012; Kurtz et al., 2015).

The abundance of microorganisms in a local community is extremely uneven, as shown by a few dominant groups playing major roles in active growth along with a large number of rare groups (Jia et al., 2018). To distinguish abundant and rare microbes in a community in terms of their roles and contributions, all OTUs detected within a community are usually divided into six exclusive categories based on relative abundance (Figure 1D; Dai et al., 2016; Chen et al., 2019c). Recently, an increasing number of studies have emphasized the importance of rare biosphere microbes, which includes more metabolically active microorganisms than abundant groups, plays a key role in co-occurrence networks, ecosystem versatility, and plant performance. These microbes are not only highly resistant to environmental stresses, but also enhance the function of abundant microbes to some extent (Jousset et al., 2017; Ziegler et al., 2018; Liang et al., 2020; Xiong et al., 2020). Similarly, some rare OMF affiliated with Serendipitaceae (such as *Serendipita indica* and *Serendipita restingae*) have been demonstrated to promote the germination of orchid seeds and the growth and adaptation of plantlets (Schäfer and Kogel, 2009; Oliveira et al., 2014; Shah et al., 2019; Fritsche et al., 2020). Interestingly, these rare OMF coexist with a wide range of plants and increase the reproduction and fitness of symbiotic hosts. Moreover, *S. indica* has value in agricultural applications due to its effect on increasing yield of tomatoes by 65%, while also inducing their resistance to salt stress (Abdelaziz et al., 2019).

Increasing attention to the mycorrhizal network of orchids, rare taxa in the network, and adaptive evolution between orchids and fungi helps identify functions of key orchid fungi and provides clues about orchid distribution, population dynamics, and the mechanisms underlying orchid–fungal mutualisms. Thus, such studies may lend insight into the following aspects with significant implications:

1. Since the protective effect of mycorrhizal symbiosis on plants is a redundant feature, some key taxa revealed by network analysis can be used as targets. The isolation and culture of these target strains can be achieved as far as possible using medium prediction techniques in combination with some characteristics of target strains (such as the increase in the proportion of rare microorganisms in acidic environments) or simulating the growth conditions of orchids in the field. In addition, the effects of their combinations on orchid germination and various growth stages can be examined to identify simplified microbial groups that dominate the community and can meet the demand for host nutrients. Tentative exploration of orchid SynCom is would be a major advancement in orchid microbiome research and a key step for the application of scientific research achievements into greenhouse and natural environments.
2. Metagenomic analysis can assist in the functional study of key species, and binning assembly with the help of contigs obtained by metagenomic splicing can help in the annotation of genes and their functions. Moreover, comparative genome analysis and evolutionary analysis of inseparable key species at the strain level may benefit from such approaches and advance our understanding of the mechanisms of ecological adaptation, nutrient mutualism, and metabolic functions of the strains.
3. Since plant roots continuously secrete carbon and other nutrients to the rhizosphere environment, the rhizosphere is known as one of the most dynamic interfaces on Earth. This significantly affects the arms race within complex microbial communities as well as the growth and health of hosts (Jogaiah et al., 2013; Raaijmakers, 2015; Lee et al., 2020). However, reports on fungal communities in the rhizosphere of orchids are currently limited. Therefore, future studies should focus on the composition, dynamics, and function of orchid fungal communities in this microdomain and the correlation of their biogeographic patterns with the distribution and population dynamics of orchids.
4. Attempts should be made to correlate the underground fungal diversity with the genetic characteristics of aboveground orchid populations to gain insight into the genetic diversity and dynamic history of orchid populations. This can be done through SSR molecular markers and ABC models or SNP markers and DADI models, to indirectly predict the dynamics of fungal diversity with the aim of protecting orchid gene pools (including orchid provenances and fungal sources).
5. Increasing evidence suggests that nitrates significantly affect the composition and abundance of OMF and inhibit the germination of orchid seeds in natural habitats (Duffy et al., 2019; Figura et al., 2020). Further work should focus on the effects of specific environmental variables, such as soil moisture content, nitrate content, and pH, which are frequently reported to affect OMF communities in network analyses, to gain insight into how these metrics affect sub-modules and the entire network. At the same time due to the lack of reports on orchid seed-associated microbial communities, we know little to nothing about the heritability of microorganisms associated with orchid species and the vertical transmission ability of the microbiome at the plant level. These are important aspects of orchid–fungus mutualism that require urgent attention.

**Figure 3:**
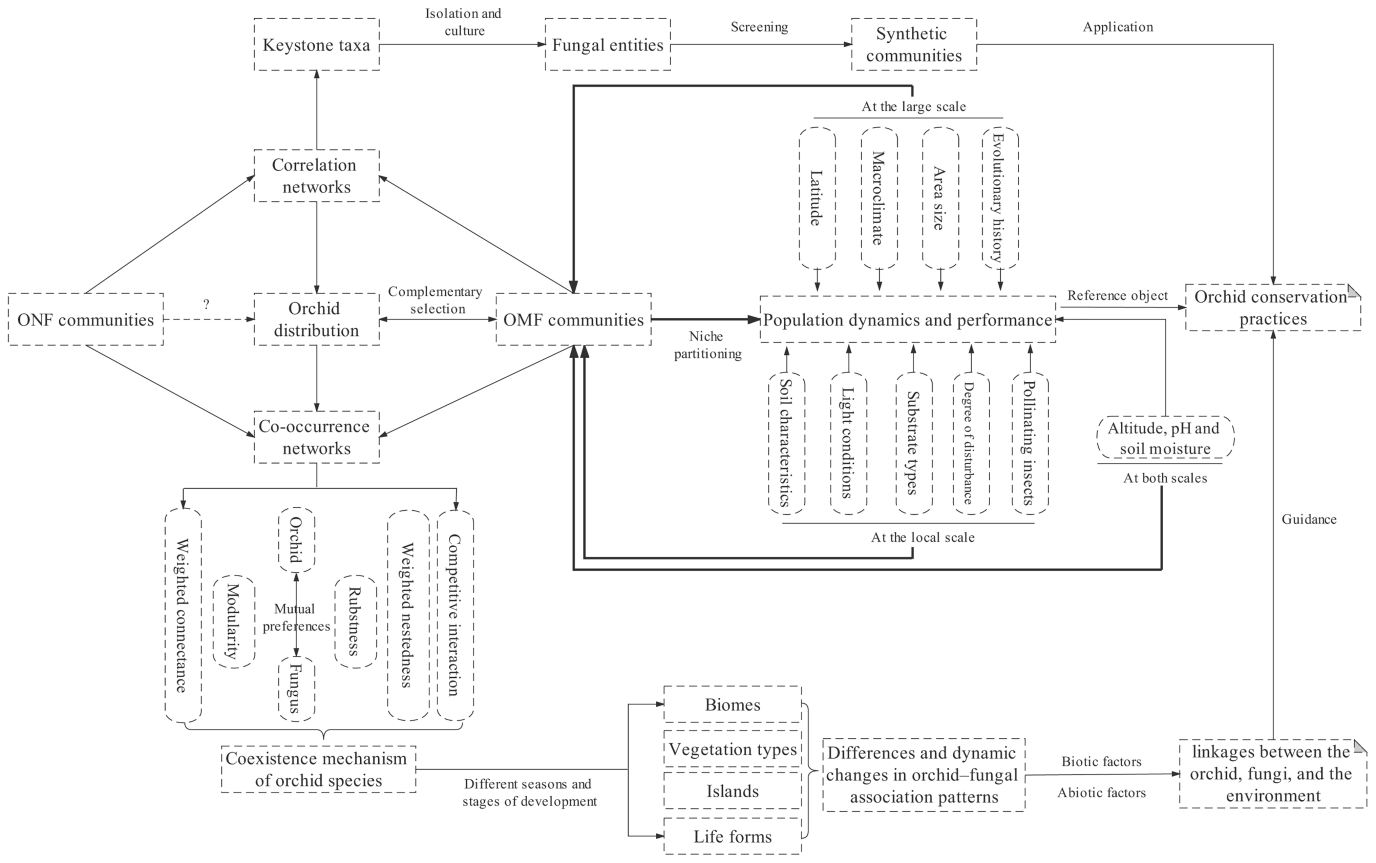
A framework illustrating that orchid fungal networks are critical for insight into the complex and dynamic linkages between the orchid, fungi, and the environment, as well as the exploration of orchid conservation practices. The thick lines imply increasing evidence that environmental factors may indirectly affect orchid distribution and population dynamics by driving niche partitioning in OMF communities (see the second paragraph in the Introduction); dashed line with question marks indicate hypothetical relationships that have rarely been studied.

Finally, in order to better point out the research direction of orchid fungal networks, while emphasizing the importance of considering ONF and making full use of network analysis methods in orchid–fungal interaction, we also provide a conceptual framework illustrating that orchid fungal networks are critical for insight into the complex and dynamic linkages between the orchid, fungi, and the environment, as well as the exploration of orchid conservation practices (Figure 3). Furthermore, several forward-looking studies have recently confirmed that certain bacterial taxa as well as microbial interkingdom interactions are essential for plant growth (Durán et al., 2018; Finkel et al., 2020). Observations under the microscope suggest that bacterial taxa mainly reside within the root caps of orchids, while fungal taxa are found in various subdivisions of roots. The spatial distribution pattern of such cross-kingdom microorganisms in roots seems to be consistent with their characteristics because fungal hyphae can act as highways for bacterial movement and also as transport systems for microorganisms belonging to other kingdoms. However, it is unclear whether microorganisms in different kingdoms engage in mutualism within the roots, and whether such mutualism affects the distribution and population dynamics of orchids. These issues should be focus areas of research on orchids and other plants in the future.

## Author Contributions

JG and M-AS designed the outline of the manuscript. TL, JG, SW, and WY collected the data and wrote the manuscript. M-AS and JG polished the article. All authors contributed to the article and approved the submitted version.

### Conflict of Interest

The authors declare that the research was conducted in the absence of any commercial or financial relationships that could be construed as a potential conflict of interest.

The reviewer FR declared a past co-authorship with one of the authors M-AS to the handling Editor.

## Supplementary Material

The Supplementary Material for this review can be found online at: https://www.frontiersin.org/articles/10.3389/fpls.2021.647114/full#supplementary-material
