# Supplementary material for: How Mycorrhizal Associations Influence Orchid Distribution and Population Dynamics
Source: Front Plant Sci. 2021 May 7;12:647114. doi: 10.3389/fpls.2021.647114 (PMC8138319; doi:10.3389/fpls.2021.647114)
Supplement: Supplementary file 1 [file Table_1.pdf]

Supplementary Information

Review title: **How Mycorrhizal Associations Influence Orchid Distribution and Population Dynamics**

Authors: ***Taiqiang Li, Shimao Wu, Wenke Yang, Marc-André Selosse and Jiangyun Gao ****

Review acceptance date: **13 April 2021**

The following Supplementary Information is available for this article:

Table S1 | The research methods, ecological premise and revealed network architecture in the "orchid mycorrhizal network" references analyzed in this review.

Life forms	Biomes	Vegetation types	Number of orchid species	Transect sizes	PCR primers	Sequencing methods	Network architecture	References
Terrestrial	Temperate	Dry calcareous grasslands through wet meadows to forests	16 species, 222 individuals	Across seven countries	ITS1OF/ITS4OF	ABI sequencing	Significantly nested (NODF = 43.88, P < 0.001)	Jacquemyn et al. (2011a)
Epiphytic+Terrestrial	Tropical	Lowland rainforests (high canopy), lowland semi-dry forests and mountain rainforests (epiphyte-rich)	77 species (50 epiphytic and 27 terrestrial species), 452 individuals	Reunion Island	ITS1F/ITS4<br>ITS1F/ITS4B<br>ITS1/ITS4Tul<br>ITS1F/TW13<br>ITS3S/TW13<br>ITS5.8S-Tul/TW13	ABI sequencing	Significantly modular (overall: M_{obs} = 0.715, P < 0.01)<br>Significantly nested (epiphytic subnetworks: NODF = 5.61, P < 0.01)	Martos et al. (2012)
Epiphytic+Terrestrial	Tropical	Epiphytic: A pristine forest and a regenerating forest site; Terrestrial: an anthropogenic influenced south facing slope	59 species	Epiphytic: 100 m^2 of pristine forest and 0.1 ha of regenerating forest; Terrestrial: not quite clear	ITS1/TW14<br>NS23/NLSeb2R<br>ITS1F/NLSeb2R<br>NS23/NLSeb1R<br>ITS1F/NLSeb1R	ABI sequencing	Significantly nested (NODF = 14.10, P = 0.001)	Kottke et al. (2013)
Terrestrial	Mediterranean	A dry Mediterranean grassland covered with sparsely distributed trees	20 species, 80 individuals	10×1000 m	ITS3/ITS4OF	454 pyrosequencing	Significantly modular (M_{obs} = 0.589, P = 0.001)	Jacquemyn et al. (2015b)
Terrestrial	Boreal and temperate	Mediterranean grasslands and forests, wetlands, peat bogs, coastal habitats and alpine-boreal habitats	14 species, 114 individuals	Across six European countries	ITS1OF/ITS4OF	454 pyrosequencing	Significantly modular (M_{obs} = 0.474, P < 0.001)	Jacquemyn et al. (2016b)
Epiphytic+Terrestrial	Tropical	A pristine forests, a 40-y-old regenerating forest and a human-caused landslide	114 individuals	56 plots of 1 m^2	ITS1/TW14	ABI sequencing	Significantly nested (NODF = 16.2, P < 0.05)	Herrera et al. (2018)

Epiphytic+Terrestrial +Mycoheterotrophic	--	--	12 orchid datasets	--	--	Meta-analysis	Significantly modular (M_{obs} = 0.377, > root endophytes and EcM fungi, P < 0.05)	Põlme et al. (2018)
Epiphytic+Terrestrial +lithophytic	Tropical	Tropical rainforests	44 species, 245 individuals	Menglun subreserve	ITS1OF/ITS4OF	ABI sequencing	Significantly modular and nested (M_{obs} = 0.739; NODF = 5.53, P < 0.05)	Xing et al. (2019)
Epiphytic	Tropical	An ancient tea estate	9 species, 45 individuals	200 hectares	ITS3/ITS4OF	Illumina MiSeq sequencing	Significantly nested (NODF = 41.59, P < 0.01)	Xing et al. (2020b)

Note: The references are consistent with the body text. Since the keyword we used in the citation network analysis was not retrieved to Kottke et al. (2013), this article is not included in Figures 1B,C of the body text. However, it is involved in the discussion of the relative importance of nestedness and modularity in orchid mycorrhizal networks.
